# Comparison of 5-Year Outcomes for Patients With Coronary Artery Disease in Groups With and Without Revascularization With Different Results of Stress Echocardiography

**DOI:** 10.4021/cr294e

**Published:** 2013-10-15

**Authors:** Angela Zagatina, Ludmila Krylova, Yuliya Vareldzhan, Tatyana V. Tyurina, Olga Clitsenko, Nadezhda Zhuravskaya

**Affiliations:** a Cardiocenter “Medika”, St. Petersburg, Russian Federation; b Leningrad Regional Cardiologic Dispensary, St. Petersburg, Russian Federation; c Northwestern Medical University n.a. I.I. Mechnikov, St. Petersburg, Russian Federation

**Keywords:** Stress echo, Exercise echo, Prognosis, Stable coronary artery disease, Outcomes after stress echocardiography

## Abstract

**Background:**

There is conflicting data in contemporary literature concerning the best way to treat patients with stable coronary artery disease; specifically, whether medical treatment alone or invasive strategies combined with medical treatment are better. The purpose of this study was to evaluate the clinical outcomes of patients with and without revascularization after stress echocardiography and to create formulas for detecting patients with a very high risk of cardiac death/major adverse cardiac event (MACE) in their present conditions.

**Methods:**

We assessed 323 patients (53.9 ± 8.4 years, 247 men), undergoing upright bicycle stress echocardiography in 2006 - 2007. During a median follow-up of 5.2 ± 0.2 years, 21 cardiovascular and 5 confirmed non-cardiac deaths occurred. Eighty-three patients underwent revascularization.

**Results:**

Stress echocardiography was normal in 32% and abnormal in 68%. All the patients with CAD were prescribed acetylsalicylic acid, statins, beta-blockers and ACE inhibitors. Eighty-seven percents of the patients took medication regularly. The percentage taking medication didn’t significantly differ in the subgroups. Two formulas were created for detecting a very high risk of cardiac death (25%) or MACE (68%) within 5 years. All the patients with abnormal stress tests were divided into two subgroups: 80 patients with revascularization and 138 subjects without revascularization. There was a significant difference in 5 year cardiac mortality if the patients had an index of wall motion abnormality (IWMA) after exercise greater than or equal to 1.3.

**Conclusion:**

It is possible to identify during stress echocardiography subjects with a very high risk for cardiac death/MACE. Patients with IWMA ≥ 1.3 had improved outcomes following revascularization.

## Introduction

Recent decades have been marked by an intensive development of pharmacologic treatment for coronary artery disease that has had an improving effect on the survival of patients with a stable form of the disease. The previous proofs of the superiority of mechanical revascularization with definitive anatomical or functional patient features date back were from the eighties and early nineties [1-3]. This has been challenged in new investigations [4]. Some modern researchers question the benefits of revascularization in stable coronary artery disease when patients are prescribed other powerful medical treatment.

The aim of our study was to analyze the 5 year outcomes of consecutive patients with stable coronary artery disease, who underwent stress echocardiography as a risk stratification tool, depending on the subsequent strategy, and to create formulas for detecting patients with a very high risk of cardiac death/major adverse cardiac events (MACE) in their present conditions.

## Methods

This is a retrospective analysis of a prospectively collected, single-center database of 580 consecutive patients who underwent a bicycle exercise echocardiography from the period of May 2006 to February 2007. Data for 323 patients were available for analysis (53.9 ± 8.4 year, 247 men). One hundred and seventy-two patients had typical angina (53%), 159 subjects had a previous myocardial infarction (49%). Two hundred and three persons had arterial hypertension (63%). Diabetes was present in 58 patients (18%).

### Exercise echocardiography

The main reasons for undergoing stress echocardiography were diagnosis and risk stratification for patients with known or suspected coronary artery disease. All the patients underwent the upright bicycle submaximal exercise test. Patients were imaged in the left lateral decubitus position before the cycle test using Sonoline G 60S (Siemens Medical Solutions, Japan). Images were obtained in the four- and two-chamber apical views, and the long- and short-axis parasternal views. The initial power output was 50 W, followed by increases of 25 W every 2 min until standard end points were reached [5]. ECG and arterial pressure monitoring were used during the test. The post-stress images, analogous to those at rest, were obtained as soon as possible after stopping the exercise, and did not occur later than 90 seconds. The images at rest and during stress were compared side by side in a cineloop display. Regional left ventricular function was assessed using a standardized 17-segment model [6] by two expert readers in 2006 - 2007 immediately after the tests. Each segment was graded on a four-point scale, with wall motion scoring 1-4 (normal wall motion scoring - 1, hypokinetic - 2, akinetic - 3, and dyskinetic - 4). Index of wall motion abnormality (IWMA) was calculated at rest and post-exercise as the sum of the scores divided by the number of segments. The changes in IWMA (dIWMA) from rest to post exercise were calculated. Patients with poor imaging were not excluded. The abnormal test was defined as when there were wall motion abnormalities before and/or after the exercise. The positive ischemical test was defined when there was an observed worsening of wall motion abnormalities, namely dIWMA> 0.

### Medical treatment

Coronary artery disease was diagnosed in 255 patients (79%) by clinical and/or exercise test data. They are all were prescribed beta-blockers, acetyl salicylic acid, statins, and ACE inhibitors. All the patients were encouraged to regularly take the medication in proper doses as was recommended by the current Guidelines [7-8].

### Follow-up and end-points

A review of the patients and their relatives were obtained by telephone or during visits to the clinic using medical records and death certificates in 2011 - 2012. The end-points were cardiac death and MACE. Cardiac death was defined as death due to acute myocardial infarction, congestive heart failure or arrhythmias. Unexpected and otherwise unexplained sudden death was also considered to be a cardiac death. Myocardial infarction was defined by accompanying medical documents.

The patients were divided into two subgroups: Subgroup 1 - with revascularization, percutaneous coronary intervention or coronary artery bypass graft surgery, Subgroup 2 - without revascularization.

### Statistics

Continuous variables were described by means and standard deviations with categorical data being expressed by percentages. For multiple comparisons ANOVA was performed. Comparison of proportions was performed with the Pearson Chi-square test and Fisher’s exact tests. Multivariate techniques (Classification Trees) were used for creating formulas to detect the high risk group. The following categorical and continuous data were accounted for in the multivariate analysis: age, height, weight, presence of arterial hypertension, functional class of angina, the fact of previous myocardial infarction and presence of diabetes; maximal physical capacity, maximal heart rate, and maximal blood pressure during exercise test, reason for stopping the exercise test, presence of significant ST depression and fact of angina during stress test; presence of wall motion abnormalities in the left descending artery, left circumflex coronary artery and right coronary artery territories before and after the exercise, IWMA, dIWMA, the presence of ischemia during exercise by echo, the fact of an abnormal test and regularity of medical treatment. The best discriminators were chosen for a high risk group, and two formulas were created for predicting cardiac death and MACE. Statistical package version 8.0 (Statistica; Stat Soft Inc., Tulsa, Oklahoma, USA) was used for statistical analysis.

## Results

### Follow-up and end-points

The follow-up was 5.2 ± 0.2 years. During follow-up 21 cardiac and 5 confirmed non-cardiac deaths occurred. Twenty cardiac deaths were confirmed by autopsy and 1 death was for causes unknown owing to the absence of relatives and a death certificate. This case was classified as cardiac without proof to the contrary. There were one hundred and eight patients with MACE: 21 cardiac deaths, 27 nonfatal myocardial infarctions and 83 patients underwent revascularization (46 coronary artery bypass grafting, 33 percutaneous coronary interventions, and 4 had both procedures performed).

### CAD group

There was 7.8% cardiac mortality within 5 years in the group with coronary artery disease, 14.2% cardiac death and/or myocardial infarction, 43% MACE. In the non-revascularized vs. revascularized subgroups frequency of death from any cause was 10.3% vs. 6.2%, P = 0.27; cardiac death was 8.8% vs. 6.2%, P = 0.48; cardiac death and/or myocardial infarction 14.7% vs. 11.1%, P = 0.44. Eighty-seven percent of patients with CAD had taken prescribed medication regularly without significant differences in the subgroups.

### Analysis of the exercise echocardiography data

**Figure 1:**
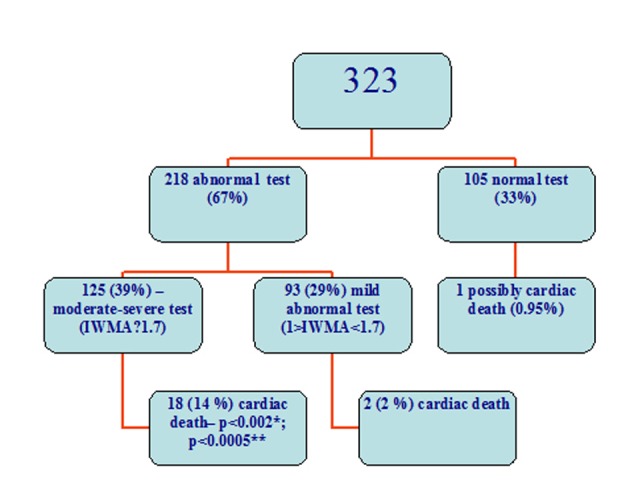
Cardiac death in the subgroups. *: difference between patients with a moderate-severe test and subjects with a mild abnormal test, **: difference between patients with a moderate severe test and a normal test.

**Figure 2:**
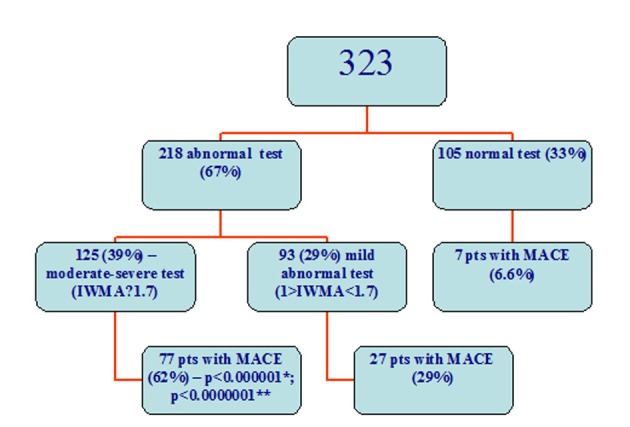
MACE in the subgroups. *: difference between patients with a moderate-severe test and subjects with a mild abnormal test, **: difference between patients with a moderate severe test and a normal test.

There were 218 abnormal and 105 normal stress echocardiography tests. Ischemia was revealed in 164 tests. All the patients were divided into groups: Group 1 with abnormal and Group 2 with normal echocardiography exercise tests. Twenty confirmed cardiac deaths occurred in the Group 1 and 1 possible cardiac death (actual cause unknown) in Group 2 (9.2 % vs. 0.95%, P < 0.006); 22 non-fatal myocardial infarctions in Group 1 and 5 in Group 2 (10.1 % vs. 4.8%, P = 0.07); 80 revascularizations were in Group 1 and 3 in Group 2 (36.7 % vs. 2.9%, P < 0.0000001); in total, 105 cases had a MACE in Group 1 and 7 in Group 2 (48.2 % vs. 6.7%, P < 0.0000001) (Fig. 1, 2).

### Subgroup with contraction abnormality/ischemia during test only by echocardiographic criteria

A total of 91 patients (42%) had abnormal tests and 47 subjects (29%) had ischemia only by echocardiographic criteria without ECG changes or angina. There were 8 cardiac deaths (9%) and 31 MACE (34%) in the subgroup of abnormal tests in patients without ECG changes or angina during exercise. There were 5 cardiac deaths (10%) and 22 MACE (47%) in the subgroup with ischemia in patients without ECG changes or angina during exercise.

### Formula for predicting high risk for 5-year cardiac death after exercise echocardiography

Multivariate analysis identified the following criteria as the strongest predictors for cardiovascular death (one of them had two cut-off points for prognosis): 1) wall motion abnormalities in the left anterior descending artery territories after exercise (HR 43.9, P < 0.0001); 2) abnormal stress echocardiography (HR 22.3, P < 0.001); 3) patient’s age greater than 70 years (HR 24.4, P < 0.01); 4) IWMA (index of wall motion abnormality) before exercise of more than 1.7 (HR 9.1, P < 0.0001); 5) wall motion abnormalities in the left descending coronary artery territories before exercise (HR 7.0, P < 0.0001); 6) wall motion abnormalities in the circumflex coronary artery territories before exercise (HR 7.0, P < 0.0001); 7) exercise capacity less than 100 WT (HR 6.1, P < 0.0005); 8) dIWMA more than 0.4 (HR 5.3, P < 0.0005); 9) wall motion abnormalities in the right coronary artery territories before exercise (HR 4.7, P < 0.005); 10) patient’s age greater than 58 years (HR 3.0, P < 0.05). The 5-year cardiovascular mortality was very high (25.3% in the group) for those with 5 to 10 of the signs noted above. The group with 0-4 points of these factors had a low mortality - 0.6%.

### Formula for predicting high risk for 5-year MACE after exercise echocardiography

Multivariate analysis identified the following criteria as the strongest predictors for MACE (two of them had two cut-off points for prognosis): 1) IWMA (index of wall motion abnormality) after exercise greater than 2.0 (HR 18.3, P < 0.0001); 2) abnormal stress echocardiography (HR 14.4, P < 0.0001); 3) a difference between IWMA before and after exercise greater than 0.3 (HR 14.3, P < 0.0001); 4) IWMA after exercise greater than 1.4 (HR 9.5, P < 0.0001); 5) wall motion abnormalities in the left anterior descending artery territories after exercise (HR 8.5, P < 0.0005); 6) angina during exercise (HR 5.2, P < 0.0005); 7) maximal heart rate during exercise lower than 120/min (HR 4.9, P < 0.0005); 8) patient’s angina functional class greater than 2 (HR 4.9, P < 0.0005); 9) wall motion abnormalities in the circumflex coronary artery territories after exercise (HR 4.7, P < 0.0005); 10) wall motion abnormalities in the right coronary artery territories after exercise (HR 4.2, P < 0.0005); 11) wall motion abnormalities in the left anterior descending artery territories before exercise (HR 4.1, P < 0.0005); 12) maximal heart rate during exercise lower than 135/min (HR 2.4, P < 0.001), 5-year MACE occurred in 7% for Group with 0-4 points, and in 68% for Group with more than 6 points.

### Revascularization

**Figure 3:**
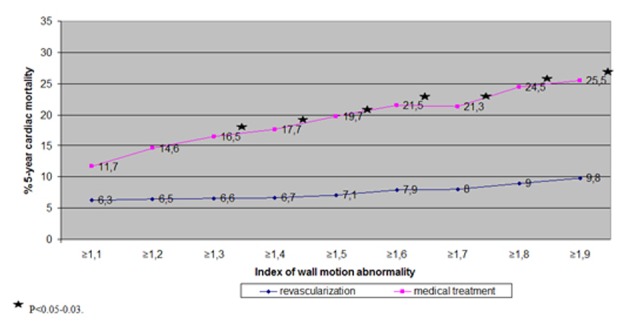
Cardiac death in the revascularized vs. non-revascularized subgroups depending on IWMA.

All the patients with abnormal stress tests were divided into two subgroups: 80 patients with revascularization and 138 subjects without revascularization. There were 3 confirmed non-cardiac deaths, their data was excluded from analysis. There occurred 5 cardiac deaths in the Subgroup 1 and 15 in the Subgroup 2 (6.3% vs. 11.1%, P = NS). However, there was a significant difference in 5-year cardiac mortality if the patients with mild abnormalities of contractility during exercise were excluded (Fig. 3). The cut-off point was 1.3 for IWMA. However in these subgroups the regularity of medical treatment was similar (93% vs. 84%, P = NS). The clinical, exercise and echocardiography characteristics of the subgroups with IWMA ≥ 1.3 with revascularization vs. without revascularization are in Table 1.

**Table 1:** Characteristics of the Subgroup With IWMA ≥ 1.3

	With revascularization N = 76	Without revascularization N = 92	P level
Age (yrs)	55 ± 8	54 ± 8	NS
Women/Men	10/66	17/75	NS
Diabetes	18 (24%)	17 (18%)	NS
AH	57 (75%)	59 (64%)	NS
MI	56 (74%)	68 (74%)	NS
Maximal HR (1/min) during exercise	121 ± 18	130 ± 18	< 0.005
WMA in LAD before exercise	43 (57%)	51 (55%)	NS
WMA in LCx before exercise	16 (21%)	29 (27%)	NS
WMA in RCA before exercise	32 (42%)	49 (53%)	NS
WMA in LAD after exercise	65 (86%)	71 (77%)	NS
WMA in LCx after exercise	47 (62%)	51 (55%)	NS
WMA in RCA after exercise	58 (76%)	66 (72%)	NS
IWMA rest	1.42 ± 0.45	1.54 ± 0.47	NS
IWMA impost	2.17 ± 0.48	1.99 ± 0.49	< 0.03
dIWMA	0.75 ± 0.40	0.45 ± 0.47	< 0.00003
Regularity of medical treatment	71 (93%)	77 (84%)	NS

AH: arterial hypertension; MI: myocardial infarction; HR: heart rate; WMA: wall motion abnormality; LAD: left anterior descending artery; LCx: left circumflex coronary artery; RCA: right coronary artery; IWMA rest: index of wall motion abnormality before exercise, IWMA impost index of wall motion abnormality immediately post exercise.

## Discussion

Stress echocardiography with its history and data spanning over 3 decades, is proving its strength in assessing patient prognosis in different groups of subjects. Generally, a normal exercise echocardiogram result is associated with an annual event rate of cardiac death and nonfatal myocardial infarction of less than 1% [9, 10]. Our study showed similar results - 4.8% non-fatal myocardial infarction in the group with normal tests within 5 years. Cardiac death was 10 times lower in this group than in the group with abnormal stress echocardiography. Likely, MACE should be further reduced in a normal test group with the implementation of non-invasive cardiac flow reserved determination, using maximal heart rate test instead of a submaximal one, using peak instead of post-exercise data etc [11-14].

In the study the subgroup with IWMA > 1.3 and without revascularization had a high cardiac mortality despite a high percentage using prescribed modern medical treatment. Cardiac mortality was incrementally increased with augmentation of IWMA and it became more than 25% in the subgroup with IWMA ≥ 1.9 if the patients had not been operated upon. The previous study had shown that cardiac mortality was not as high in patients with a stable form of coronary artery disease which was being medically treated. In these cases, it was about 0.9-1.6% annually, 4.5-8% over 5 years [4, 8]. This data was observed for all patients without dividing them into subgroups according to risk. In our study if the mortality was calculated for the entire group of patients with diagnosed coronary artery disease by clinically and/or stress test data, then there would also not be a very high cardiac mortality. This would be in spite of fact that our consecutive subjects probably suffered more severe cases of heart disease than what is present in the general population. It’s known that patients selected from a hospital setting have more severe forms of a condition precisely because they are being hospitalized. Naturally, subjects with a less severe form of disease are treated in an outpatient d. There were no significant differences in the group as a whole regarding types of strategy - revascularization or not-revascularization. Only patients with signs of poor prognosis may have had improved survival rates following an operation. So we cannot conclude, as do some of the contemporary studies, that patients with a stable form of coronary disease with modern medical treatment have a benign prognosis comparable with revascularization. There is a large group of patients with poor prognosis without revascularization and non-invasive stress echocardiography test remains able to provide valuable information regarding which patients should be selected for invasive treatment.

In the study we created formulas for detecting patients with a very high risk of cardiac death/major adverse cardiac events (MACE) in their present conditions. They are easy to use and contain only the patient’s characteristics and their stress echocardiography data, which is not time consuming to obtain or interpret. They are practical for detecting patients with a 25% 5-year risk of cardiac death, and with a 68% risk of MACE.

### Limitations

This work has all the limitations of a cohort, single-center study.

There were not a large number of patients, but the number of patients was sufficient for significant differences in the subgroups and for arriving at the main conclusions.

The experts of a single center assessed the stress echograms. As it is known, stress echocardiography is subjective and highly dependent on the specialist’s qualification method [15].

The main group was not divided into subgroups with different types of revascularization. This was outside of the goals of this study.

### Conclusion

Patients with moderate to severe contraction abnormality during exercise echocardiography tests have a high 5-year cardiac mortality despite prescribed medical treatment.

It is possible to identify during stress echocardiography the subjects with very high risk for MACE using formulas utilizing the characteristics of a patient and their test data.

Revascularization can improve 5-year survival of patients with IWMA ≥ 1.3 during exercise echocardiography in their present conditions.

## Declaration

None declare.
